# Operational Gaps in Implementing the COVID-19 Case Investigation and Contact Tracing in Madhesh Province of Nepal, May–July 2021

**DOI:** 10.3390/tropicalmed7060098

**Published:** 2022-06-10

**Authors:** Nishant Thakur, Florian Vogt, Srinath Satyanarayana, Divya Nair, Krishna Garu, Koshal Chandra Subedee, Shrawan Kumar Mandal, Amrit Pokhrel, Dipendra Gautam, Krishna Prasad Paudel

**Affiliations:** 1 Epidemiology and Disease Control Division, Department of Health Services, Kathmandu 44600, Nepal; krishnagaru1@gmail.com (K.G.); koshalsubedee@gmail.com (K.C.S.); pokhrelashu@gmail.com (A.P.); 2 National Centre for Epidemiology and Population Health, The Australian National University, Canberra 2601, Australia; florian.vogt@anu.edu.au; 3 The Kirby Institute, University of New South Wales, Sydney 2052, Australia; 4 Center for Operational Research, International Union against Tuberculosis and Lung Disease, 75001 Paris, France; ssrinath@theunion.org (S.S.); divya.nair@theunion.org (D.N.); 5 International Union against Tuberculosis and Lung Disease, New Delhi 110016, India; 6 Sukraraj Tropical and Infectious Disease Hospital, Kathmandu 44600, Nepal; fusion7722@gmail.com; 7 Country Office Nepal, World Health Organization, Kathmandu 44600, Nepal; gautamd@who.int; 8 Ministry of Health and Population, Kathmandu 44600, Nepal; kpkalyan@gmail.com

**Keywords:** COVID-19, Nepal, case investigation, contact tracing, key performance indicators, operational research (SORT IT), challenges, gaps

## Abstract

In Nepal, case investigation and contact tracing (CICT) was adopted as an important public health measure to reduce COVID-19 transmission. In this study, we assessed the performance of CICT in Madhesh Province of Nepal against national benchmarks, using routine programmatic data reported by district CICT teams. Between May and July 2021, 17,943 COVID-19 cases were declared in the province, among which case investigation was performed for 30% (95% CI: 29.6–31.0%) within 24 h (against 80% benchmark). As a result of case investigations, 6067 contacts were identified (3 contacts per 10 cases), of which 40% were traced and tested for SARS-CoV-2 infection (against 100% benchmark). About 60% of the contacts tested positive. At most 14% (95% CI: 13.1% to 14.9%) of traced contacts underwent a 14-day follow-up assessment (against 100% benchmark). We found the performance of the CICT program in Madhesh Province to be sub-optimal and call for corrective measures to strengthen CICT in the province and the country at large. Similar studies with wider geographical scope and longer time frames are needed to identify and address deficiencies in data recording and reporting systems for COVID-19, in low- and middle-income countries like Nepal and others.

## 1. Introduction

Nepal reported its first case of COVID-19 on 23 January 2020, and by the end of March 2022 the country had reported more than 1,119,271 cases with 11,952 deaths [1]. There were wide geographic and non-linear variations in the number of COVID-19 cases reported in the country during this period, due to the complex transmission dynamics, surveillance and public health measures undertaken to control the spread of the disease [2].

Immediately after COVID-19 was declared a Public Health Emergency of International Concern (PHEIC) by the World Health Organization (WHO) on 30 January 2020, several measures were adopted to detect and contain the spread of COVID-19 cases in the country [3,4]. The most prominent measure was the implementation of case investigation and contact tracing (CICT) [3]. The CICT program was developed by the Nepal’s Epidemiology and Disease Control Division (EDCD)—the focal agency for International Health Regulation in the country [5]. For implementing CICT, health worker teams were formed at local municipality level. The CICT program involved investigating confirmed cases to identify their contacts, tracing and testing those contacts, and depending on the COVID-19 test results, either isolating or quarantining the contacts to halt further transmission.

Several studies around the world have shown that CICT, when implemented optimally, can be a successful public health intervention to stop the transmission of infectious diseases [6,7]. CICT helps to identify, assess, and manage people at risk of having contracted infection and becoming infectious [8]. For CICT to be effective, it is critical to measuring its performance against certain benchmarks through well-defined and relevant indicators. Several frameworks provide such performance indicators [8]; broadly these indicators relate to inputs, processes, outputs, outcomes and impact [9]. Failing to assess the performance of CICT can lead to gaps in surveillance, with detrimental effects for public health.

In Nepal, during the initial stages of the CICT implementation, data was scarce due to poor uptake of Go.data—an online real-time recording and reporting system that was introduced by Nepal’s EDCD with support from the WHO [10]. To address this poor uptake, EDCD developed a simplified data recording and reporting format using Microsoft Excel^®^ and established a national secretariat to oversee the implementation of CICT in May 2021. This recording and reporting system involved manually gathering CICT data from district level health worker teams known as CICT teams, and then aggregating and transmitting the data to the secretariat in a structured Microsoft Excel^®^ reporting format. Nepal experienced a rise in COVID-19 cases due to the delta variant (from mid-April to end of July 2021), and the secretariat received CICT reports from all districts. These data were compiled at the secretariat but were not systematically analyzed to understand the performance of the CICT program against certain benchmarks.

Therefore, we undertook an operational research study in one of the provinces (Madhesh Province) of Nepal to describe the performance of the CICT and identify areas of the recording and reporting system that require improvement. The specific objectives were to assess (a) the numbers of COVID-19 cases recorded in the CICT systems between mid-April and July 2021, (b) the proportion of cases investigated within 24 h, and of contacts traced within 48 h, and the completeness of the data collection forms used for CICT and (c) the status of COVID-19 testing of close contacts.

## 2. Materials and Methods

### 2.1. Study Design

This was a cross-sectional study involving secondary analysis of routine programmatic data.

### 2.2. Setting

#### 2.2.1. General Setting

Nepal is a land-locked country situated between India and China, with an area of 147,181 square kilometers and a population of 30 million (as of 2022). Nepal’s health service delivery is primarily a mixed model, with private institutions constituting large part of curative services whereas preventive and promotive services are usually delivered by the government and supporting agencies. The Department of Health Services (DoHS) governs and manages the organization of health services in Nepal, and EDCD is the division of DoHS assigned for framing and implementing public health policies and progammes for communicable and non-communicable disease control. 

#### 2.2.2. Specific Setting

Nepal has seven provinces, together comprising of 77 districts and 753 municipalities. Most of the public health programs in Nepal are implemented at district level, through district health offices. These offices are headed by chief district health officers who implement different health programs at a municipality level. Madhesh Province is situated in south-eastern part of the country and has eight districts: Bara, Parsa, Mahottari, Dhanusha, Sarlahi, Saptari, Rautahat, and Siraha. District health offices became the designated local bodies for implementation and supervision, of the CICT program, and for reporting its results, and they formed health worker teams for undertaking CICT activities. Virtual and in-person training was given to build their capacity to conduct CICT.

#### 2.2.3. The CICT Program

**Figure 1:**
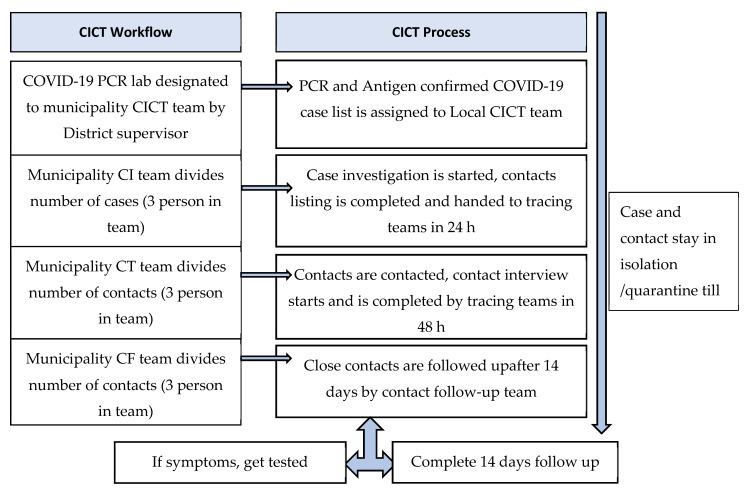
Work process of CICT system in Nepal.

After the declaration of COVID-19 as a PHEIC by the WHO in 2020, a technical team was formed, and standard operating procedures were developed with technical assistance of the WHO Nepal Country Office. The process of CICT is described in Figure 1. CICT involves case interviews and tracing all newly confirmed cases within 24 h and documenting their close contacts through a case investigation form (A form), tracing and testing close contacts for COVID-19 infection and documenting their status using a close contact form (B1 form) within 48 h of the index case notification and isolating close contacts if they test positive. If they test negative, they are quarantined for 14 days, followed up, and the outcome documented using a follow-up form (B2 form) on the 14th day. In the Nepalese CICT system, cases are those who are laboratory confirmed (through nucleic acid amplification tests and/or rapid antigen tests), and contacts refer to people who are within 2 m proximity of a confirmed case for more than 15 min. The A forms, B1 forms and B2 forms were completed as hard paper copies by the CICT teams and summaries of daily activities were reported electronically using Google sheets (created using Microsoft Excel^®^), updated every 24 h.

### 2.3. Study Population and Study Period

Our study population were laboratory-confirmed COVID-19 cases and their close contacts identified through the CICT reporting system between May and July 2021 in Madhesh Province. The province was selected for this study because the principal investigator was involved with supervising and monitoring the data collection process from EDCD in this province.

### 2.4. Data Variables and Sources of Data

The data variables included aggregated district-wide information on the daily number of COVID-19 cases enrolled into the CICT, the number of cases who underwent investigation, and the respective numbers of contacts identified, traced, tested and followed up. The data source was the daily report submitted online on Google sheet created using Microsoft Excel^®^ (created by Microsoft Corporation, Redmond, WA, USA) from each district of Madhesh Province. 

### 2.5. Analysis and Statistics

Each district within the province was considered as a unit of analysis. The data was summarized using numbers, proportions, and averages. The following performance indicators and their benchmarks were used for assessing the performance of the CICT program:

o At least 80% of the cases are investigated within 24 h of reporting.
o 100% of the ‘A forms’ are filled for all cases investigated.
o At least 80% of the contacts are traced and interviewed within 48 h, and 100% of the contacts traced are tested for COVID-19 infection.
o 100% of the ‘B2 forms’ are completed for all contacts traced.

We operationally defined ‘A forms’ and ‘B2 forms’ to be completed if all data fields on the respective forms pertaining to cases and their contacts were completed.

Missing data were observed in ~1% of the data fields and have been excluded from the analysis and reported as missing. The data summary measurements [average, rates, proportions, and confidence interval (CI)] were calculated in Microsoft Excel^®^, 2013.

## 3. Results

### 3.1. Case Investigation

Overall, between May and July 2021, 17,943 COVID-19 cases were reported through the CICT system in Madhesh province. The average daily new cases registered was 213 (minimum 5, maximum 605) (Table 1). Among the eight districts in the province, Dhanusha district recorded the maximum average daily new cases (63 cases/day) and Saptari district recorded the fewest average daily new cases (11 cases/day). 

**Figure 2:**
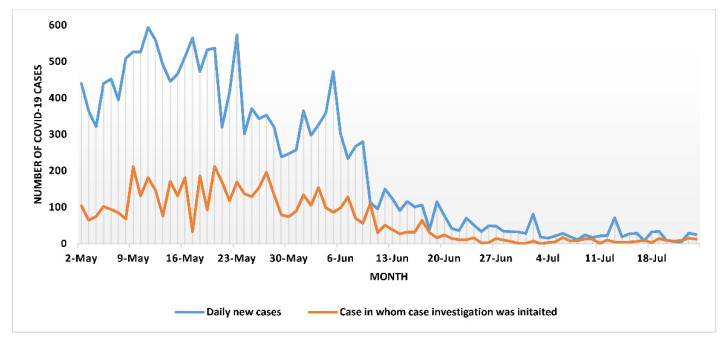
Daily trend of new COVID-19 cases and initiation of case investigation among cases reported to the CICT program between May to July 2021 in province-2 of Nepal.

At the province level, the CICT teams-initiated case investigation for 30.3% (95% CI: 29.6–31.0%) of the cases within 24 h of case notification, with Mahottari district initiating the highest (50.8%) and Siraha district initiating the lowest proportion of cases (9.5%) (Table 1). The gap between cases notified and investigated was highest during the initial part of the CICT program, when the daily case load was high compared to the later part of the study period (Figure 2). Overall, ‘A forms’ pertaining to case investigation were found to be complete for 18.2% (95% CI: 17.7–18.8%) of the cases investigated. The proportion of ‘A forms’ found to be complete ranged from 40.2% in Rautahat district to 4.2% in Bara district (Table 1).

### 3.2. Contact Tracing

Overall, 6067 close contacts were identified during case investigation in the province (Table 2). The highest number of contacts were in Dhanusha district (2592) and the lowest number of contacts were found in Sarlahi district (132). At the province level there were on an average about 3 contacts per 10 cases investigated, with the highest in Rautahat district (7 contacts per 10 cases investigated) and the lowest in Siraha and Sarlahi districts (1 contact per 10 cases investigated).

During the study period, average contacts to be traced per day at the province level was 73 contacts, with Dhanusha district having to trace on average 31 contacts per day, and Siraha and Sarlahi districts each having to trace on average 2 contacts per day.

### 3.3. COVID-19 Testing of Contacts

Of the contacts traced at the province level, 40% (95% CI: 38.7–41.2%) were tested for COVID-19 infection within the study period May to July 2021. The proportion tested ranged from 54% in Sarlahi district to 16% in Siraha and Rautahat districts (Table 2). At the province level, 64% (95% CI: 62.7–65.2%) of those tested were found to be positive for COVID-19 infection. The positivity rate was highest in Para district (100%) and lowest in Bara district (27%). Overall, 14% of the ‘B2 contact tracing forms’ (describing the contact status at the end of 14-day follow-up) were found to be complete in all aspects. In Sarlahi district 97% of the B2 forms were found to be complete when compared to 0% of the forms in Bara district. As the reporting formats provided data only on the number of B2 contact tracing forms completed and not on the status of the contacts at the end of the 14-day follow-up period, we were unable to describe the status of contacts (infected, not infected etc.) at the end of 14 days.

## 4. Discussion

This is the first study from the WHO’s SEARO region to assess the performance of CICT against pre-defined key indicators. Our study showed that during May to July 2021, about a third of COVID-19 cases were investigated within 24 h of being notified, with on average three contacts for every 10 cases investigated. About 40% of the contacts were traced and tested for COVID-19 with a positivity rate of 60%. The 14-day follow-up was completed for about 14% of the contacts who were traced and tested.

The strength of this study is that it was conducted using routine programmatic data and hence the findings reflect ground-level realities. The study has a few limitations. First, the study was conducted in one conveniently sampled province of Nepal, and therefore the study findings may not be generalizable to other provinces of the country. Conducting similar analysis in other provinces will help overcome this limitation. Second, the study was conducted using aggregate data at the district level, as information on CICT at the level of individual cases and contacts was difficult to obtain. Hence, we are unable to describe case/contact characteristics associated with inadequate CICT performance. Third, due to resource and time constraints, we could not gather information on challenges associated with using routine data for assessing CICT performance against key indicators from all stakeholders. Gathering information on challenges in generating, collecting and using routine program data to make inferences about CICT performance would have provided more comprehensive information on how to improve this program and is an area for future research. Despite these limitations, the study findings have certain implications on policy and practice.

First, during the study period only 30% of the cases were investigated within 24 h, against the benchmark of at least 80%. The gaps in the case investigation were highest when the daily caseload was high. The possible reasons for this underperformance are perhaps insufficient human resources (CICT teams) rather than neglect or hesitancy by the CICT teams to conduct the activity. Anecdotal evidence also indicates that in several instances, case information was provided to the CICT teams after 24 h of diagnosis and the peripheral CICT teams prioritized conducting case investigation for those diagnosed within the last 24 h, and neglected others. Allocating more human resources and ensuring case information reaches CICT teams in a timely manner is essential for improving the case investigation process.

Second, in our study three contacts were identified per 10 cases investigated (i.e., less than one contact per case) and there were huge inter-district variations in the contacts identified per case. The number of contacts identified per case is lower than studies conducted in other parts of world that have reported on average 3.0 to 4.6 contacts per case [11]. The reasons why the contacts were low in our setting is unknown and is an area for future investigation. Anecdotal evidence indicates possible challenges in identifying contacts who meet the operational definition of a close contact, and the inability of individuals to recollect close contact information. In addition, several individuals mentioned isolating themselves immediately if they developed symptoms or if they learned that anyone whom they had contacted developed COVID-19. Also, physical distancing and wide-spread stay-at-home orders were in effect [12] which reduced close contact between people. At this stage, we feel that assessment of the reasons for fewer contacts per case in our setting requires further investigation.

Third, only 40% of the contacts were tested for COVID-19 infections, against the benchmark of 100%, and about 60% of those tested were found to be COVID-19 positive. The testing rate and positivity rate are comparable to other studies in the South Asia region [13,14]. The reasons for low testing rates include refusal to undergo testing due to contacts being asymptomatic, shortages or non-availability of antigen rapid diagnostic tests for point-of-care testing, and difficulties in transporting contacts or their samples to health facilities where rt-PCR testing was available. Educating contacts about the importance of timely testing will improve higher uptake of COVID-19 testing among contacts, and ensuring adequate quantities of point-of-care antigen rapid diagnostic tests is vital.

Fourth, the ‘B2 form’ that provides data on the 14-day status of contacts was completed for only 14% of contacts, against the benchmark of 100%. This is perhaps due to the inability of the peripheral health teams to perform this activity, secondary to human resource constraints, or non-prioritization of this activity by the CICT teams [15]. Failures to complete the 14-day follow-up of contacts have also been highlighted in several low-income countries [16]. Non-availability of ‘B2 forms’ curtailed our ability to make inferences on the effectiveness of the CICT process; the lack of information on the status of contacts at the end of 14 days makes it difficult to judge how far it contributed to preventing spread of the disease and providing early diagnosis for provision of timely care.

Fifth, several gaps in the recording and reporting formats were identified by the co-authors. Approximately 1% of the cells did not contain any data, and missing data can introduce bias in assessing the performance of the CICT program. The current reporting formats provided information on aggregate numbers of cases investigated in 24 h, contacts traced etc., daily. These formats captured information on the activities performed on any given day. With this information it is difficult to make comprehensive inferences on the performance of the case investigation and contact tracing. Nevertheless, the available information indicates that the performance of the CICT program was less than satisfactory. Therefore, there is an urgent need to review and revise the recording and reporting formats (including development or adoption of existing digital applications) to strengthen the CICT program in the country, through a multistakeholder consultative process involving the district teams involved in conducting CICT. This will help in obtaining more robust information on CICT program performance and enable timely corrective measures.

The study highlights the importance of operational research in assessing the performance of CICT processes. Several similar interventions were undertaken internationally for preventing infection, early diagnosis, and prompt treatment of COVID-19. This study can act as a role model and stimulate interest and encourage others to undertake similar assessments, to evaluate whether the public health measures being undertaken to contain COVID-19 infection are having the desired impact.

## 5. Conclusions

The performance of the CICT program in Madhesh Province, Nepal, was sub-optimal and fell short against the national benchmark indicators. The CICT recording and reporting formats require revision to provide actionable information on key performance indicators. There is an urgent need to assess challenges and develop solutions for better implementation of CICT in Nepal and other low- and middle-income countries in general.

## Figures and Tables

**Table 1:** District trends in timeliness of initiation and completion of case investigation in the CICT program in Province-2 of Nepal, May to July 2021.

Districts	Reported Cumulative Cases	Average Daily Caseload (Range)	Case Investigation Initiated within 24 h * % (95% CI)	Case Investigation Completed % (95% CI) **
**Overall (Madhesh Province)**	**17,943**	**213 (5–605)**	**30.3 (29.6–31.0)**	**18.2 (17.7–18.8)**
Dhanusha	5307	63 (0–218)	40.6 (39.2–41.9)	34.1 (32.8–35.4)
Mahottari	2566	30 (0–112)	50.0 (48.1–52.0)	12.8 (11.6–14.2)
Parsa	2400	29 (0–163)	20.8 (19.1–22.4)	4.5 (3.8–5.4)
Bara	1952	23 (0–236)	12.6 (11.2–14.2)	4.2 (3.4–5.2)
Saptari	1888	11 (0–119)	18.4 (16.6–20.2)	9.1 (7.8–10.4)
Siraha	1580	19 (0–92)	9.5 (8.1–11.0)	9.2 (7.8–10.7)
Rautahat	1277	15 (0–93)	44.4 (41.6–47.1)	40.2 (37.5–42.9)
Sarlahi	973	12 (0–73)	20.3 (17.9–23.1)	11.8 (9.8–14.0)

* National benchmark for case investigation initiation is 80%. ** case investigation completed = Proportion of new cases for which A forms were completed.

**Table 2:** District-wide variations in identification, testing, positivity rates and completion of 14-day follow-up for contacts via the CICT program in Province-2 of Nepal between May to July 2021.

Districts	Contacts Identified	Average Contacts Per 10 Cases Investigated	Average Daily Contact Load	Contacts Tested % (95% CI)	Positivity Rate among Contacts Tested % (95% CI)	Contact Assessments Completed * % (95% CI)
**Overall (Province 2)**	**6067**	**3**	**73**	**40 (38.7–41.2)**	**64 (62.7–65.2)**	**14 (13.1–14.9)**
Dhanusha	2592	5	31	19 (17.4–20.5)	50 (48.0–51.9)	7 (6.0–7.9)
Mahottari	466	2	6	100	59 (54.5–63.4)	14 (10.9–17.1)
Parsa ^#^	1200	5	14	38 (35.2–40.7)	100	9 (7.3–10.6)
Bara	382	2	5	36 (31.9–40.8)	27 (22.5–31.4)	0
Saptari ^$^	364	2	4	-	-	33 (28.1–37.8)
Siraha	137	1	2	16 (9.8–22.1)	73 (65.5–80.4)	39 (30.8–47.1)
Rautahat	794	7	10	16 (13.4–18.5)	91 (89.0–92.9)	24 (21.0–26.9)
Sarlahi	132	1	2	54 (45.5–62.5)	45 (36.5–53.4)	97 (94.0–99.9)

CICT—case investigation and contact tracing. * contact assessment was considered competed if 14 day follow up was completed and a B2 form completed. ^#^ Parsa district’s CICT reporting form reported the same number for total contacts tested and contacts that tested positive. ^$^ Saptari district omitted from analysis of testing and positivity rate due to data discrepancies.

## Data Availability

The data from this available upon request from the principal investigator.

## Disclaimer

In accordance with WHO’s open-access publication policy for all work funded by WHO or authored/co-authored by WHO staff members, WHO retains the copyright of this publication through a Creative Commons Attribution IGO license (http://creativecommons.org/licenses/by/4.0/igo/legalcode, accessed on 7 June 2022) which permits unrestricted use, distribution and reproduction in any medium provided the original work is properly cited. There should be no suggestion that WHO endorses any specific organization, products or services. The views expressed in this article are those of the authors and do not necessarily reflect those of their affiliated institutions. The use of the WHO logo is not permitted. This notice should be preserved along with the article’s original URL.

## References

[1] Nepal Daily Situation Report of COVID-19 in Nepal. http://heoc.mohp.gov.np/.

[2] Li Z., Jones C., Ejigu G.S., George N., Geller A.L., Chang G.C., Adamski A., Igboh L.S., Merrill R.D., Ricks P. (2021). Countries with Delayed COVID-19 Introduction-Characteristics, Drivers, Gaps, and Opportunities. Glob. Health.

[3] Nepal Health Research Council Rapid Assessment of COVID-19 Related Policy Audit in Nepal. http://nhrc.gov.np/wp-content/uploads/2021/02/Policy-audit-Report-Final-MD.pdf.

[4] Piryani R.M., Piryani S., Shah J.N. (2020). Nepal’s Response to Contain COVID-19 Infection. J. Nepal Health Res. Counc..

[5] Epidemiology and Disease Control Division A Guide to Early Warning and Reporting System (EWARS). https://www.edcd.gov.np/resources/download/a-guide-to-ewars-2019.

[6] Spencer K.D., Chung C.L., Stargel A., Shultz A., Thorpe P.G. (2021). COVID-19 Case Investigation and Contact Tracing Efforts from Health Departments-United States, June 25–July 24, 2020. Morb. Mortal. Wkly. Rep..

[7] Keeling M.J., Hollingsworth T.D., Read J.M. (2020). Efficacy of Contact Tracing for the Containment of the 2019 Novel Coronavirus (COVID-19). J. Epidemiol. Community Health.

[8] Center for Disease Control Evaluating Case Investigation and Contact Tracing Success. https://www.cdc.gov/coronavirus/2019-ncov/php/contact-tracing/contact-tracing-plan/evaluating-success.html.

[9] Vogt F., Kurup K.K., Mussleman P., Habrun C., Crowe M., Woodward A., Jaramillo-gutierrez G., Kaldor J., Vong S., Vilas R. (2022). Contact Tracing Indicators for COVID-19: Rapid Scoping Review and Conceptual Framework. PLoS ONE.

[10] Bhatt N., Bhatt B., Gurung S., Dahal S., Jaishi A.R., Neupane B., Budhathoki S.S. (2020). Perceptions and Experiences of the Public Regarding the COVID-19 Pandemic in Nepal: A Qualitative Study Using Phenomenological Analysis. BMJ Open.

[11] Lash R.R., Moonan P.K., Byers B.L., Bonacci R.A., Bonner K.E., Donahue M. (2021). COVID-19 Case Investigation and Contact Tracing in the US. JAMA Netw. Open.

[12] Awale S. (2020). Kathmandu Locks Down Again. https://www.nepalitimes.com/latest/kathmandu-locks-down-again/.

[13] Koetter P., Pelton M., Gonzalo J., Du P., Bogale K., Buzzelli L., Connolly M., Katelyn Edel A.H., Nicole R., Legro D.M. (2020). Implementation and Process of a COVID-19 Contact Tracing Initiative: Leveraging Health Professional Students to Extend the Workforce During a Pandemic. Am. J. Infect. Control.

[14] Kretzschmar M.E., Rozhnova G., Bootsma M.C.J., van Boven M., van de Wijgert B. (2020). Impact of Delays on Effectiveness of Contact Tracing Strategies for COVID-19: A Modelling Study. Lancet Public Health.

[15] Eyawo O., Viens A.M. (2021). Lockdowns and Low- and Middle-Income Countries: Building a Feasible, Effective, and Ethical COVID-19 Response Strategy. Glob. Health.

[16] Lewis D. (2020). Why Many Countries Failed at COVID Contact-Tracing--but Some Got It Right. Nature.

